# Broadband zero-backward and near-zero-forward scattering by metallo-dielectric core-shell nanoparticles

**DOI:** 10.1038/srep12491

**Published:** 2015-08-18

**Authors:** Yan Li, Mingjie Wan, Wenyang Wu, Zhuo Chen, Peng Zhan, Zhenlin Wang

**Affiliations:** 1 School of Physics and National Laboratory of Solid State Microstructures, Nanjing University, Nanjing 210093, China; 2 Collaborative Innovation Center of Advanced Microstructures, Nanjing University, Nanjing 210093, China

## Abstract

Efficient control of optical radiation at subwavelength scales plays important roles for various applications. Dielectric nanoparticles or dielectric shells with a large refractive index of *n* ~ 3–4, which are only achievable for limited semiconductors, are involved in most designs so far to control the scattering by overlapping the electric and magnetic dipolar modes of the same magnitude. Here we propose a new mechanism based on the interplay between dipolar and quadrupolar resonances of different amplitudes, both magnetic and electric, to suppress the backward scattering or the forward scattering by using metallo-dielectric core-shell nanoparticles with a dielectric shell layer having a refractive index of *n* = 2.0. We demonstrate that broadband zero-backward or near-zero-forward scattering can be achieved by optimizing the structural parameters. We also demonstrate that the core-shell nanoparticles with identical dielectric shells but metal cores with various sizes are able to suppress the backward or forward scattering at the same wavelength, thus revealing a large tolerance to fabrication errors induced by the size distributions in the metal cores. These features make the proposed core-shell nanoparticles beyond the dipole limit more easily realized in practical experiments.

Light scattering by small particles has long been a topic of great interest to researches in many directions from optical communications, astrophysics to biophysics and material science^1^,^2^,^3^,^4^. Among different research fields related to the particle scattering, efficient control of optical radiation at subwavelength scales, e.g. suppressing the unwanted backward scattering (BS) and enhancing the directional forward scattering (FS), is one of the most crucial issues and plays important roles for various applications, such as nanoantennas^5^,^6^, sensors^7^,^8^, light-emitting devices^9^ and photovoltaic devices^10^. It has been theoretically predicted long ago that the scattered field by a magnetic sphere exhibiting both electric and magnetic dipolar resonances could be controlled with coherent effects between both dipoles^11^. Under certain conditions for the values of relative electric permittivity (*ε*) and relative magnetic permeability (*μ*) of the magnetic sphere, its response to plane-wave illumination may consist of electric and magnetic dipoles with equal amplitudes^11^, in which the in-phase and out-of-phase oscillation of the dipoles could lead to the zero-backward (first Kerker condition) and near-zero-forward (second Kerker condition) radiated power, respectively^12^. Although the proposed coherent effects between electric and magnetic resonances supported by magnetic spheres could control the scattered radiation more flexibly without involving complicated structures^11^,^13^,^14^, compared with the pure electric-response-based approach for manipulation of the scattering patterns, where the complicated structures are usually involved^5^,^6^,^15^,^16^,^17^,^18^,^19^,^20^,^21^,^22^, they were thought to be impossible to be realized in the visible region because natural substances exhibit negligible magnetism at optical frequencies, that is, their relative magnetic permeability is unity (*μ* = 1)^23^.

Recently, it has been realized that the emerging concept of artificial optical magnetism demonstrated in the engineered metamaterials could tackle the aforementioned issue. Various metamaterial structures, such as split-ring-resonator^24^, silver nanoparticle trimer^25^, high-permittivity dielectric nanoparticles^12^,^26^,^27^,^28^,^29^,^30^,^31^,^32^,^33^,^34^,^35^, metallo-dielectric and all-dielectric core-shell geometries^36^,^37^,^38^,^39^,^40^, have been theoretically and experimentally investigated for scattering control. In most of these studies, the suppression of the BS is achieved by satisfying the first Kerker condition within the dipole limit^12^,^26^,^27^,^28^,^29^,^30^,^31^,^32^,^33^,^36^,^37^. This approximation requires dielectric nanoparticles or dielectric shells with a large index of refraction (*n* ~ 3–4), which is only achievable for limited semiconductor materials, such as silicon, germanium, and gallium arsenide^12^,^26^,^27^,^28^,^29^,^30^,^31^,^32^,^33^,^36^,^37^. For example, for a small high-permittivity particle within the dipole limit, the electric (*a*_*1*_) and magnetic (*b*_*1*_) dipole terms of Mie expansion^1^ dominantly contribute to the scattered field, while the higher-order terms are normally negligible (*a*_*l*_ = *b*_*l*_ = 0, *l* ≥ 2). Therefore, the first Kerker condition is expected to be easily satisfied by only engineering the electric and magnetic dipoles so as to satisfy *a*_*1*_ = *b*_*1*_^26^. By embedding a metallic nanoparticle into a high-permittivity nanosphere, a simultaneous suppression of BS and enhancement of FS can be achieved in the resonant superscattering regime^33^,^36^,^37^, in which the high-permittivity shell supports a strong magnetic dipolar response, and can be tuned to overlap spectrally with the electric dipolar resonance of the metal core^41^. Furthermore, it has been shown that higher order electric and magnetic modes are able to play an important role in controlling the directivity of scattered radiation^24^,^34^,^35^, but their excitations require specific dipole sources^33^. More recently, by extending the case of overlapping electric and magnetic dipoles of the same magnitude to higher order modes, it has been theoretically demonstrated that simultaneously ultra-directional FS and suppressed BS can be achieved in rationally designed core-shell nanoparticles with incident plane waves^39^.

Here we show that metallo-dielectric core-shell nanoparticles, consisting of a silver core and a dielectric shell layer with a refractive index of *n* = 2.0, can exhibit a broadband zero-backward scattering (ZBS) beyond the dipole limit. In contrast to the first Kerker condition that requires equal electric and magnetic multipole coefficients (*a*_*l*_ = *b*_*l*_)^11^, our proposed mechanism is based on the interplay between dipolar and quadrupolar Mie resonances, both electric and magnetic, having different amplitudes and satisfying the condition of 3(*a*_1_ − *b*_1_) = 5(*a*_2_ − *b*_2_), and thus has no particular requirement of high-permittivity materials. We demonstrate that although the BS is suppressed in the off-resonant scattering regimes, the enhanced unidirectional FS can still be achieved due to the involvement of the contributions from magnetic and electric quadrupolar Mie terms. At a certain selected wavelength, the ZBS is found to be insensitive to the variation of the core radius, revealing a relatively large tolerance to the size distributions in the metal cores. A dependence of the spectral position of the broadband ZBS on the shell thickness is also demonstrated, providing an easy and precise way to tune the ZBS to the desired wavelength. Similarly, based on the coherent effects between dipolar and quadrupolar Mie resonances, we further demonstrate that a broadband near-zero-forward scattering (NZFS) can also be realized in the proposed metallo-dielectric core-shell nanostructures when the real and imaginary parts of the Mie term 3(*a*_1_ + *b*_1_) + 5(*a*_2_ + *b*_2_) simultaneously reach minima.

## Results

**Figure 1:**
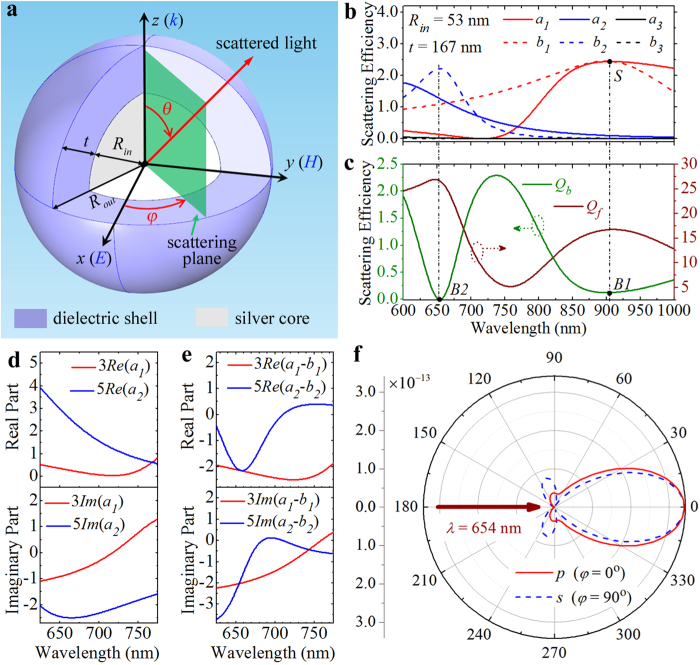
Scattering properties of a single-layered core-shell nanoparticle. (**a**) Geometry of a core-shell nanostructure and associated coordinate system. The incident plane wave is polarized along the *x*-direction and propagates along the *z*-direction. The scattered light is specified by the scattering angle *θ* and the azimuthal angle *φ*. The silver core has a radius of *R*_*in*_ = 53 nm. The dielectric shell has a refractive index of *n* = 2.0 and a thickness of *t* = 167 nm. [Note: The graph is drawn by Zhuo Chen.] (**b**) Scattering spectra of the first three electric *a*_1_, *a*_2_, *a*_3_ (solid curves) and the first three magnetic *b*_1_, *b*_2_, *b*_3_ (dashed curves) multipolar contributions. Marked point *S* at the wavelength of *λ* = 906 nm indicates that the electric and magnetic dipole terms have equal coefficients (*a*_*1*_ = *b*_*1*_). (**c**) BS (olive curve) and FS (wine curve) efficiencies versus the incident wavelengths. Marked point *B1*, corresponding to the point *S* in (**b**), indicates a non-zero BS efficiency at the wavelength of *λ* = 906 nm. Marked point *B2* indicates a zero BS efficiency at the wavelength of *λ* = 654 nm. (**d**) Real and imaginary parts of the Mie terms 3*a*_1_ and 5*a*_2_. (**e**) Real and imaginary parts of the dipole-related Mie term 3(*a*_1_ − *b*_1_) and the quadrupole-related term 5(*a*_2_ − *b*_2_). (**f**) 2D scattering patterns calculated at the wavelength of *λ* = 654 nm on the scattering planes of the azimuthal angles *φ* = 0° (red solid curve) and 90° (blue dashed curve).

Light scattering by a spherical particle in free space can be solved analytically using Mie theory^1^. A schematic view of the single-layered core-shell nanoparticle and associated coordinate system under study are shown in Fig. 1a. The silver core has a radius of *R*_*in*_, and the thickness of the coated concentric dielectric shell is *t*. Throughout the paper, the permittivity of silver is taken from the experimental data of Johnson and Christy^42^, and the refractive index of the dielectric shell is assumed to be *n* = 2.0. The incident plane wave is assumed to be polarized along the *x*-direction and propagate along the *z*-direction. The scattered light is specified by the scattering angle *θ* (the angle from the incident direction) and the azimuthal angle *φ* which uniquely determines the scattering plane defined by the incident direction and the scattering direction (see Fig. 1a). The total scattering efficiency *Q*_*sca*_ defined as scattering cross section divided by the cross section of the particle is^1^:


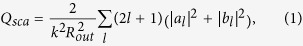


where *k* is the wavenumber, *R*_*out*_ = *R*_*in*_ + *t* is the outer radius of the core-shell structure, *a*_*l*_ is the *l*-th order transverse-magnetic (TM or electric) Mie scattering coefficient, and *b*_*l*_ is the *l*-th order transverse-electric (TE or magnetic) Mie scattering coefficient. The BS efficiency (*Q*_*b*_) and the FS efficiency (*Q*_*f*_), which respectively corresponds to the scattering efficiency at the backward (*θ* = 180°) and forward (*θ* = 0°) directions, are^1^:


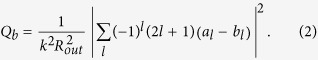



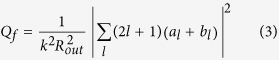


We first characterize the separate contributions of different multipole modes for a concentric spherical core-shell nanoparticle with a metallic core of radius *R*_*in*_ = 53 nm and a dielectric shell of thickness *t* = 167 nm. Figure 1b shows the calculated scattering efficiencies of the dipolar terms (*a*_*1*_, *b*_*1*_), quadrupolar terms (*a*_2_, *b*_2_), and octupolar terms (*a*_3_, *b*_3_). It is clearly seen that within the displayed wavelength range from 600 nm to 1000 nm, both the electric and magnetic-based dipolar and quadrupolar terms of Mie expansion dominantly contribute to the scattered fields, while the higher-order octupolar terms are negligible (see Fig. 1b). This confirms that for such core-shell structures with a relatively larger outer radius of *R*_*out*_ = 220 nm, the dipole approximation is invalid. Furthermore, even when the electric (*a*_*1*_) and magnetic (*b*_*1*_) dipole terms have equal coefficients (marked point *S* in Fig. 1b, corresponding to a wavelength of *λ* = 906 nm), the first Kerker condition is not satisfied due to the attendance of unequal electric and magnetic quadrupolar terms (*a*_*2*_ ≠ *b*_*2*_), leading to a non-zero BS efficiency (marked point B1 in Fig. 1c). Interestingly, it is observed that at a shorter wavelength of *λ* = 654 nm (marked point B2 in Fig. 1c) BS is found to be completely suppressed. Here, it should be noted that in this case the first Kerker condition is still not satisfied, because the electric and magnetic dipolar terms have different coefficients, as well as the quadrupolar terms (*a*_*1*_ ≠ *b*_*1*_ and *a*_*2*_ ≠ *b*_*2*_, see Fig. 1b).

To better understand the ZBS observed at the wavelength of *λ* = 654 nm, we only keep the dipolar (*a*_*1*_, *b*_*1*_) and quadrupolar terms (*a*_2_, *b*_2_) in Eq. (2), because the scattered fields can be well described by using these four Mie terms (see Fig. 1b). Under this approximation, the BS efficiency (*Q*_*b*_) and the FS efficiency (*Q*_*f*_) can be written as^12^:


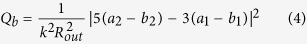



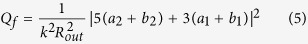


From a mathematical point of view, two particular solutions (*a*_*1*_ = *b*_*1*_, *a*_*2*_ = *b*_*2*_) and (3*a*_*1*_ = 5*a*_*2*_, 3*b*_*1*_ = 5*b*_*2*_) can make Eq. (4) equal to zero (*Q*_*b*_ = 0). Clearly, since the first Kerker condition^11^ is not satisfied in our case (*a*_*1*_ ≠ *b*_*1*_ and *a*_*2*_ ≠ *b*_*2*_, see Fig. 1b), the former particular solution *a*_*1*_ = *b*_*1*_ and *a*_*2*_ = *b*_*2*_ is excluded. Although the later particular solution 3*a*_*1*_ = 5*a*_*2*_ and 3*b*_*1*_ = 5*b*_*2*_ has previously been suggested to be able to suppress the BS totally^39^, it should also be excluded in our case. To demonstrate this, the real and imaginary parts of the Mie terms 3*a*_*1*_ and 5*a*_*2*_ are plotted in Fig. 1d as functions of the wavelength. It is clearly seen from Fig. 1d that neither the real nor the imaginary parts of 3*a*_*1*_ and 5*a*_*2*_ are equal at the wavelength of *λ* = 654 nm (corresponding to the point B2 where the BS efficiency is zero, see Fig. 1c), i.e., 3*a*_*1*_ ≠ 5*a*_*2*_. Therefore, the only possibility to satisfy the equation of *Q*_*b*_ = 0 in our case is the dipole-related term 3(*a*_1_ − *b*_1_) and the quadrupole-related term 5(*a*_2_ − *b*_2_) are identical. Figure 1e shows the real and imaginary parts of these two terms, from which it is immediately found that at the wavelength of *λ* = 654 nm the real parts of the dipole- and quadrupole-related terms indeed match and their imaginary parts are equal as well. Meanwhile, the FS efficiency (*Q*_*f*_) is also calculated according to Eq. (5) and shown in Fig. 1c. It is seen that at *λ* = 654 nm the FS efficiency reaches a maximum value of ~27. This confirms that although the BS is suppressed in the off-resonant scattering regimes (see Fig. 1b), the enhanced FS can still be achieved due to the contributions from both dipole and quadrupolar terms.

In order to give a full characterization of the scattering by the core-shell nanoparticles, we further investigate the angular distribution of the far-field normalized scattered intensity (NSI, normalized to the incident intensity). Again, due to the negligible contributions of octupolar and higher-order Mie coefficients, it is sufficient to include only the dipolar and quadrupolar Mie coefficients in the polarized components of the scattered irradiance, which are then expressed as^12^,^36^:


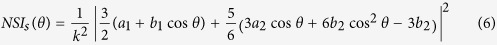


if the incident light is polarized perpendicular to the scattering plane, and


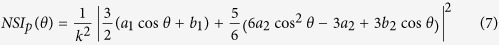


if the incident light is polarized parallel to the scattering plane. Figure 1f shows the two-dimensional (2D) scattering patterns calculated at *λ* = 654 nm on the scattering planes of the azimuthal angles *φ* = 0° (*xz*-plane in Fig. 1a, corresponding to the *p*-polarized component) and *φ* = 90° (*yz*-plane in Fig. 1a, corresponding to the *s*-polarized component). According to Eqs. (6) and (7), the scattered irradiance in the backward direction *θ* = 180° can be simplified as


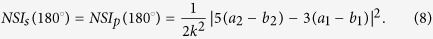


As has been demonstrated above, at the specified wavelength of *λ* = 654 nm, the dipole-related term 3(*a*_1_ − *b*_1_) is equal to the quadrupole-related term 5(*a*_2_ − *b*_2_) (see Fig. 1e). According to Eq. (8), the scattering by the proposed core-shell structure in the backward direction is exactly zero, as proven in Fig. 1f. Furthermore, it is seen that most of the scattered energy is radiated into the forward hemisphere with a relatively small angular beamwidth (the full width at half maximum of the NSI) of ~60°, indicating a good directionality. Here, it should be noted that due to the complex interplay between dipolar and quadrupolar electric and magnetic resonances of different amplitudes, there exist other possible scattering angles *θ* at which *s*- and *p*-polarized components reach a local minimum or a local maximum, thus leading to extra side scattering lobes^39^. For example, at *θ* ≈ 63° (or 297°) and 105° (or 255°) the *s*-polarized component reaches local maxima and local minima, respectively (see blue dashed curve in Fig. 1f). Except for those local maxima, the scattering has been effectively suppressed in the whole backward hemisphere (90° < *θ* < 270°). The above analyses unambiguously confirm the possibility of producing the ZBS effect and therefore the enhancement of the directional FS by exploiting the interplay between dipolar and quadrupolar Mie coefficients of different amplitudes in a core-shell nanostructure with a low-refractive-index dielectric shell, in which no particular requirement of the satisfaction of the first Kerker condition is needed.

**Figure 2:**
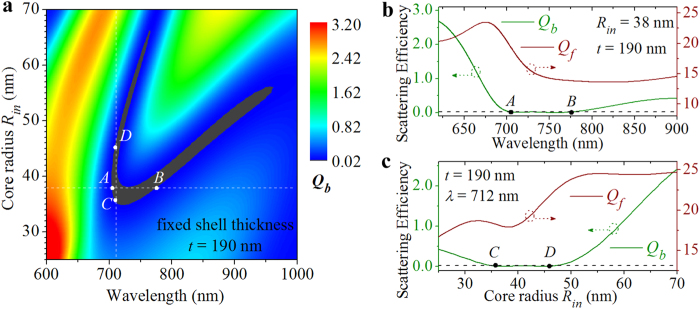
BS efficiency evolution of a core-shell nanoparticle with different core sizes. (**a**) Contour plot of the BS efficiency as a function of the incident wavelength and the core radius. The shell thickness is fixed to *t* = 190 nm. Dark-greyish colored area represents low BS efficiency less than 0.02. Marked points *A* (*R*_in_ = 38 nm, *λ* = 705 nm) and *B* (*R*_in_ = 38 nm, *λ* = 776 nm) define a 70-nm-wide ZBS in the horizontal dimension. Marked points *C* (*R*_in_ = 36 nm, *λ* = 712 nm) and *D* (*R*_in_ = 46 nm, *λ* = 712 nm) define a 10-nm-wide ZBS in the vertical dimension. (**b**) BS (olive curve) and FS (wine curve) efficiencies versus the incident wavelengths for a core-shell nanoparticle with a core radius of *R*_in_ = 38 nm and a shell thickness of *t* = 190 nm, corresponding to the special case indicated by a horizontal dashed line in (**a**). (**c**) BS (olive curve) and FS (wine curve) efficiencies versus the core radius are shown at the incident wavelength of *λ* = 712 nm, corresponding to the special case indicated by a vertical dashed line in (**a**).

In the following, we demonstrate that it is possible to achieve a relatively broadband ZBS by optimizing the silver core radius (*R*_*in*_). Figure 2a shows the BS efficiencies (*Q*_*b*_) for the core-shell nanoparticles with a fixed shell thickness of *t* = 190 nm as a function of the wavelength and the core radius. In the calculations, the refractive-index of the dielectric shell is still assumed to be *n* = 2.0. It should be noted that the complete suppression of BS can only be achieved at certain wavelengths where the condition of 3(*a*_1_ − *b*_1_) = 5(*a*_2_ − *b*_2_) is satisfied. From a practical point of view, the BS efficiency at very low levels (*Q*_*b*_ < 0.02) can be approximated to the ZBS, as is the case for the rest of the paper. For clarity, the ZBS in Fig. 2a is represented by a dark-greyish colored area. It is clearly seen that the ZBS only appear within the core radius range of 35 nm ≤ *R*_*in*_ ≤ 66 nm and the wavelength range of 703 nm ≤ *λ* ≤ 958 nm, and forms a sickle-shaped region. As indicated by a horizontal dashed line in Fig. 2a, ZBS is achieved at the core radius of *R*_in_ = 38 nm, and covers the spectrum range of wavelengths from *λ* = 705 nm (marked point *A*) to *λ* = 776 nm (marked point *B*). In addition to the broadband ZBS in the horizontal (wavelength) dimension, the sickle-shaped region provides a broadband ZBS in the vertical (core radius) dimension. As indicated by a vertical dashed line in Fig. 2a, the BS is found to be always suppressed at the particular wavelength of *λ* = 712 nm, as long as the core radius is within the range between *R*_*in*_ = 36 nm (marked point *C*) and *R*_*in*_ = 46 nm (marked point *D*). Corresponding to the above two special cases, both the BS and FS efficiencies are plotted in Fig. 2b,c as functions of the wavelength (for a core radius of *R*_*in*_ = 38 nm) and as functions of the core radius (for a wavelength of *λ* = 712 nm), respectively. It is directly seen that the BS efficiency spectra within the AB and CD regions (olive curves in Fig. 2b,c) are well below the black dashed line representing the value of 0.02. Meanwhile, the FS within these two regions are found to maintain a relatively large efficiency above the value of 14 (wine curves in Fig. 2b,c).

**Figure 3:**
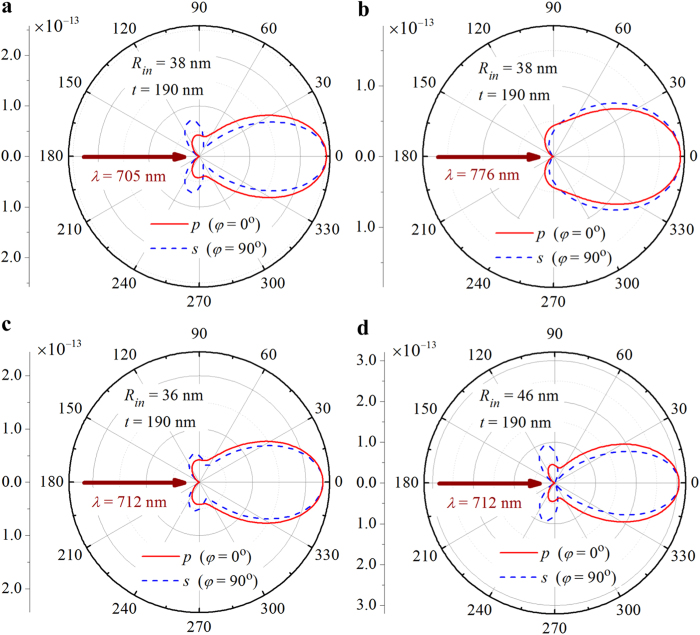
2D scattering patterns at the ZBS. (**a**) and (**b**) Scattering patterns are calculated for the same core-shell nanoparticle (*R*_in_ = 38 nm, *t* = 190 nm) but at different wavelengths of *λ* = 705 nm and *λ* = 776 nm, respectively. (**c**) and (**d**) Scattering patterns are calculated at the same wavelength of *λ* = 712 nm for core-shell nanoparticles with identical shell thickness of *t* = 190 nm but different core radii of *R*_in_ = 36 nm and *R*_in_ = 46 nm, respectively.

The 2D scattering patterns are further calculated at marked points *A* (*λ* = 705 nm and *R*_*in*_ = 38 nm, Fig. 2a) and *B* (*λ* = 776 nm and *R*_*in*_ = 38 nm, Fig. 2a), and displayed in Fig. 3a,b, respectively. It is found that the scattering in the backward direction is almost completely suppressed at both points, demonstrating a 71 nm bandwidth of the ZBS for the core-shell nanoparticles with a core radius of *R*_*in*_ = 38 nm and a shell thickness of *t* = 190 nm. It is also seen that only small part of the scattered energy is radiated into the backward hemisphere (90° < *θ* <270°) at the scattering angles within the side scattering lobes for both *s-* (dashed curves in Fig. 3a,b) and *p*-polarized (solid curves in Fig. 3a,b) components. Most of the scattered energy is radiated into the forward hemisphere with an angular beamwidth of ~60° at point A (Fig. 3a) and ~80° at point B (Fig. 3b), indicating a good directionality. Figures 3c,d show, respectively, the 2D scattering patterns at marked points *C* (*λ* = 712 nm and *R*_*in*_ = 36 nm) and *D* (*λ* = 712 nm and *R*_*in*_ = 46 nm), which have similar features to those shown in Fig. 3a,b. This confirms that the suppression of the BS with concomitant unidirectional FS can be achieved within a 10-nm-wide core radius range from 36 to 45 nm for a given wavelength of *λ* = 712 nm.

**Figure 4:**
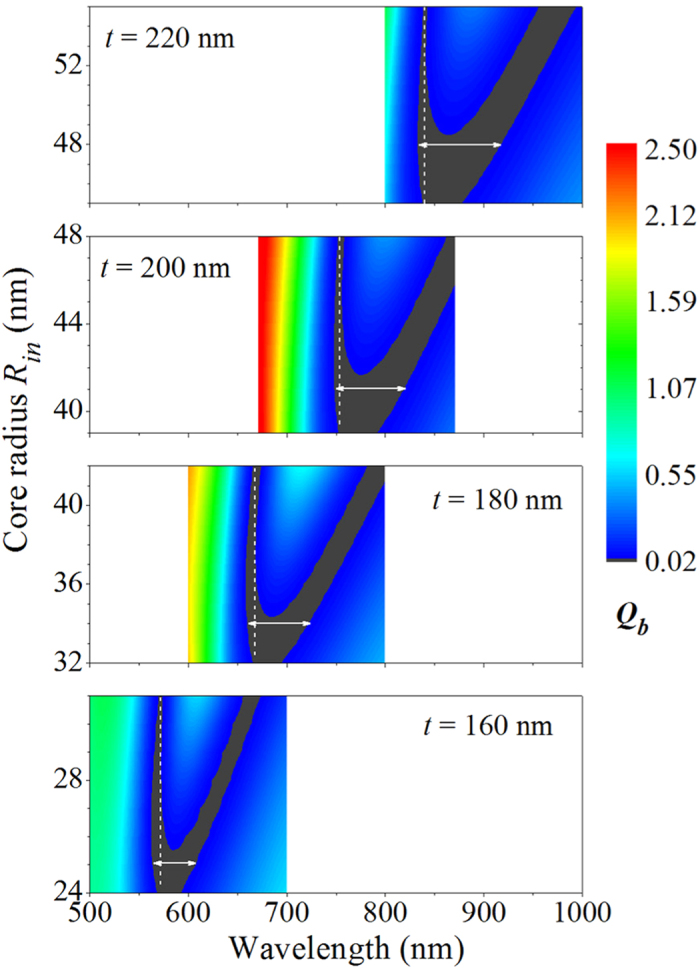
Structural tunability of the broadband ZBS. Contour plots of the BS efficiency as a function of the incident wavelength and the core radius for different shell thicknesses. From the bottom panel to the top panel, the shell thickness increases from *t* = 160 nm to *t* = 220 nm. The solid horizontal arrow and dashed vertical line in each panel indicates the broadband ZBS in the horizontal (wavelength) and vertical (core radius) dimensions, respectively.

In the frame of Mie theory, the scattering coefficients are dependent on the size parameter^1^, which makes it possible to tune the ZBS to the desired wavelength by varying the geometrical parameters. Actually, it has been demonstrated in Fig. 2a that for the core-shell nanoparticles with a fixed shell thickness of *t* = 190 nm the variation of the core radius allows the ZBS to be tuned from 704 nm to 960 nm. Here, we only focus on the structural tunability of the broadband ZBS. To demonstrate this, the *Q*_*b*_ for core-shell nanoparticles with four different shell thicknesses of *t* = 160, 180, 200, and 220 nm are plotted in Fig. 4 as a function of the wavelength and the core radius. It is clearly seen that in each case there is a sickle-shaped ZBS region which preserves the broadband nature in both the horizontal (wavelength) and vertical (core radius) dimensions. For a smaller shell thickness of *t* = 160 nm, a broadband ZBS in the horizontal dimension with a bandwidth of 43 nm centered at the visible wavelength of *λ* ≈ 586 nm can be observed at the core radius of *R*_in_ = 25 nm, as indicated by a horizontal solid arrow in the bottom panel of Fig. 4. Meanwhile, a 7-nm-wide ZBS centered at the core radius of *R*_in_ ≈ 28 nm is found in the vertical dimension for the wavelength of *λ* = 572 nm, as indicated by a vertical dashed line in the bottom panel of Fig. 4. Comparing all the cases shown in Fig. 4, it is obvious that the sickle-shaped ZBS region gradually shifts to the longer wavelength and larger core radius upon increasing the shell thickness. In this way, when the shell thickness increases to *t* = 220 nm (see the top panel of Fig. 4), the broadband ZBS in the horizontal dimension with an increased bandwidth of 84 nm is found to be achieved at a larger core radius of *R*_in_ = 48 nm, and its central wavelength red-shifts to the near infrared wavelength of *λ* = 875 nm. At the same time, the broadband ZBS in the vertical dimension for the shell thickness of *t* = 220 nm also shifts to a longer wavelength of *λ* = 840 nm and covers the range of core radii from 45 nm to 55 nm.

**Figure 5:**
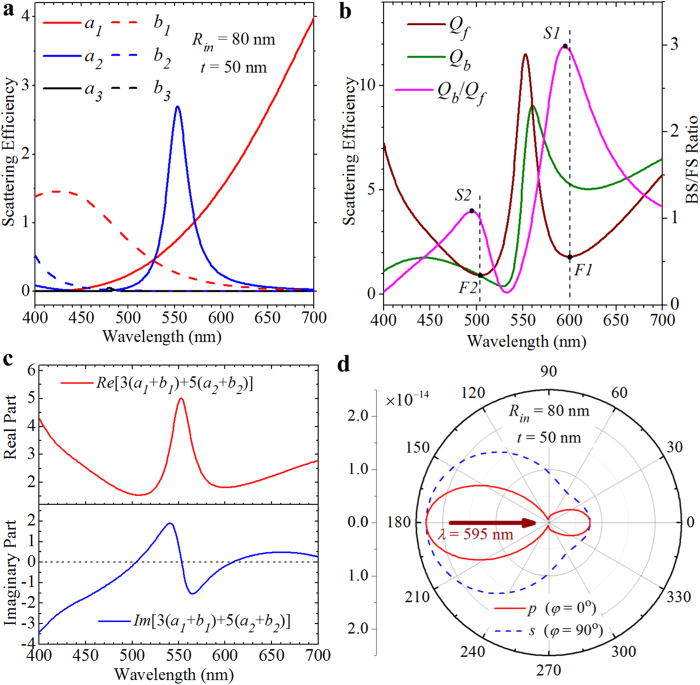
NZFS of a single-layered core-shell nanoparticle. (**a**) Scattering spectra of the first three electric *a*_1_, *a*_2_, *a*_3_ (solid curves) and the first three magnetic *b*_1_, *b*_2_, *b*_3_ (dashed curves) multipolar contributions calculated for a core-shell nanosphere with a silver core of radius *R*_*in*_ = 80 nm and a dielectric shell of thickness *t* = 50 nm. (**b**) BS efficiency (olive curve), FS efficiency (wine curve) and the ratio of the BS efficiency to the FS efficiency (magenta curve) versus the incident wavelengths. Marked points *F1* and *F2* indicate two FS efficiency minima located at the wavelengths of *λ* = 600 nm and *λ* = 654 nm, respectively. Marked points *S1* and *S2* indicate the wavelengths of *λ* = 595 nm and *λ* = 495 nm, respectively, where the BS/FS ratio reaches maximum values. (**c**) Real and imaginary parts of the Mie term 3(*a*_1_ + *b*_1_) + 5(*a*_2_ + *b*_2_). (**d**) 2D scattering patterns calculated at the wavelength of *λ* = 595 nm, corresponding to the marked point *S1* in (**b**).

In addition to the ZBS demonstrated above, NZFS is also possible to be achieved by using the proposed metallo-dielectric core-shell nanostructures. Figure 5a shows the calculated scattering efficiencies of the first three electric (*a*_*1*_, *a*_*2*_, *a*_*3*_) and magnetic (*b*_*1*_, *b*_*2*_, *b*_*3*_) Mie terms for a concentric spherical core-shell nanosphere with a silver core of radius *R*_*in*_ = 80 nm and a dielectric shell of thickness *t* = 50 nm. Again, it is seen that within the displayed wavelength range from 400 nm to 700 nm, the scattered fields can be well described by using the electric dipolar (*a*_*1*_), magnetic dipolar (*b*_*1*_), electric quadrupolar (*a*_*2*_) and magnetic quadrupolar (*b*_*2*_) Mie terms. Therefore, the BS efficiency (*Q*_*b*_) and FS efficiency (*Q*_*f*_) can be calculated using Eqs (4) and (5), and the results are shown in Fig. 5b. Two minima with values of *Q*_*f*_ = 1.8 and 0.9 are found to be located at the wavelengths of *λ* = 600 nm (marked point *F1* in Fig. 5b) and *λ* = 504 nm (marked point *F2* in Fig. 5b) in the FS efficiency spectrum, respectively. In previous studies^11^,^12^,^30^, the NZFS (the second Kerker condition) has been achieved within the dipole limit, where the real parts of electric (*a*_*1*_) and magnetic (*b*_*1*_) dipolar terms match, and their imaginary parts are equal but with different sign. Here, it should be noted that the second Kerker condition is not satisfied in our case due to the involvement of higher-order quadrupolar terms (see Fig. 5a). Actually, Eq. (5) can get a minimum value when both the real and imaginary parts of the Mie term 3(*a*_1_ + *b*_1_) + 5(*a*_2_ + *b*_2_) reach minima. Figure 5c shows the real and imaginary parts of this Mie term, from which it is confirmed that around the wavelengths of *λ* = 600 nm and *λ* = 504 nm its imaginary part indeed approaches to zero and its real part simultaneously gets a minimum value.

It is also seen from Fig. 5b that around the wavelength of *λ* = 600 nm (marked point *F1*) the BS efficiency (*Q*_*b*_ ≈ 5.4) is much larger than the FS efficiency (*Q*_*f*_ ≈ 1.8), while the BS efficiency (*Q*_*b*_ ≈ 1.0) is quite close to the FS efficiency (*Q*_*f*_ ≈ 0.9) around the wavelength of *λ* = 504 nm (marked point *F2*). As a result, at the wavelengths of *λ* = 595 nm (marked point *S1* in Fig. 5b) and *λ* = 495 nm (marked point *S2* in Fig. 5b), which are slightly shorter than the wavelengths where the NZFS is achieved, the ratio of the BS efficiency to the FS efficiency (*Q*_*b*_/*Q*_*f*_) reaches two maxima with values of ~3.0 and ~1.1, respectively. Figure 5d shows the 2D scattering patterns of the *p*-polarized and *s*-polarized components calculated at *λ* = 595 nm (corresponding to the marked point S1 in Fig. 5b). It is seen that most of the scattered energy is radiated into the backward hemisphere with a relatively small angular beamwidth of ~48° for the *p*-polarized component and a relatively large angular beamwidth of ~106° for the *s*-polarized component.

**Figure 6:**
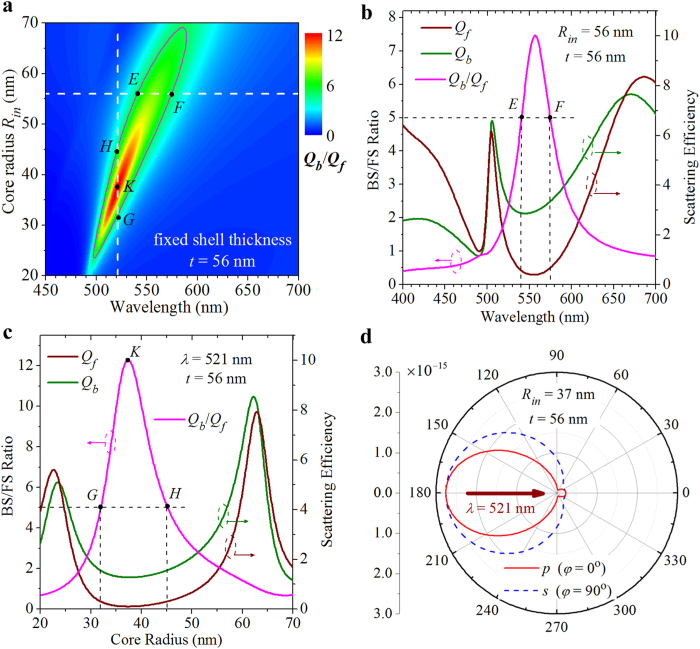
BS/FS ratio evolution of a core-shell nanoparticle with different core sizes. (**a**) Contour plot of the BS/FS ratio as a function of the incident wavelength and the core radius. The shell thickness is fixed to *t* = 56 nm. Solid black line outlines the boundary at which the BS/FS ratio equals 5. Marked points *E* (*R*_in_ = 56 nm, *λ* = 541 nm) and *B* (*R*_in_ = 56 nm, *λ* = 574 nm) define a 33-nm-wide NZFS with BS/FS ratio higher than 5 in the horizontal dimension. Marked points *G* (*R*_in_ = 32 nm, *λ* = 521 nm) and *D* (*R*_in_ = 45 nm, *λ* = 521 nm) define a 13-nm-wide NZFS in the vertical dimension. Marked point *K* (*R*_in_ = 37 nm, *λ* = 521 nm) indicates that the BS/FS ratio reaches a value as high as ~12. (**b**) BS/FS ratio (magenta curve), BS efficiency (olive curve), and FS efficiency (wine curve) versus the incident wavelengths for a core-shell nanoparticle with a core radius of *R*_in_ = 56 nm and a shell thickness of *t* = 56 nm, corresponding to the special case indicated by a horizontal dashed line in (**a**). (**c**) BS/FS ratio (magenta curve), BS efficiency (olive curve), and FS efficiency (wine curve) versus the core radius are shown at the incident wavelength of *λ* = 521 nm, corresponding to the special case indicated by a vertical dashed line in (**a**). (**d**) 2D scattering patterns calculated for a core-shell nanoparticle with a core radius of *R*_in_ = 37 nm and a shell thickness of *t* = 56 nm at the wavelength of *λ* = 521 nm, corresponding to the marked point *K* in (**a**) and (**c**).

Furthermore, we demonstrate that it is possible to further suppress the FS and achieve a relatively broadband high BS/FS ratio by optimizing the silver core radius and the dielectric shell thickness. Figure 6a shows the BS/FS ratios (*Q*_*b*_/*Q*_*f*_) for the core-shell nanoparticles with a fixed shell thickness of *t* = 56 nm as a function of the wavelength and the core radius. For clarity, the solid black line is used in Fig. 6a to outline the boundary at which the BS efficiency is 5 times as large as the FS efficiency (*Q*_*b*_/*Q*_*f*_ = 5). As indicated by a horizontal dashed line in Fig. 6a, high BS/FS ratio (*Q*_*b*_/*Q*_*f*_ ≥ 5) is achieved at the core radius of *R*_in_ = 56 nm, and covers the spectrum range of wavelengths from *λ* = 541 nm (marked point *E*) to *λ* = 574 nm (marked point *F*). In addition to the broadband high BS/FS ratio in the horizontal (wavelength) dimension, the region bounded with the solid black line provides a broadband high BS/FS ratio in the vertical (core radius) dimension. As indicated by a vertical dashed line in Fig. 6a, the BS/FS ratio is found to be above 5 (*Q*_*b*_/*Q*_*f*_ ≥ 5) at the particular wavelength of *λ* = 521 nm, as long as the core radius is within the range between *R*_*in*_ = 32 nm (marked point *G*) and *R*_*in*_ = 45 nm (marked point *H*). Corresponding to the above two cases, the BS/FS ratios are plotted in Fig. 6b,c as functions of the wavelength (for a core radius of *R*_*in*_ = 56 nm) and as functions of the core radius (for a wavelength of *λ* = 521 nm), respectively. It is directly seen that the BS/FS ratios within the *EF* and *GH* regions are well above the black dashed horizontal line representing the value of 5. Meanwhile, the BS and FS efficiencies are also plotted in Fig. 6b,c. It is found that the FS efficiency is below the value of 0.65, while the BS is dominant and maintains a relatively large efficiency above the value of 2.9 within the *EF* region (see Fig. 6b). Within the entire *GH* region, the FS and BS efficiencies are well below the value of 0.31 and above the value of 1.3, respectively (see Fig. 6c). In particular, it is found that for a core-shell nanoparticle with a core-radius of *R*_*in*_ = 37 nm and a shell thickness of *t* = 56 nm the FS efficiency goes below 0.1 at the wavelength of *λ* = 521 nm (marked point *K* in Fig. 6a and Fig. 6c). Although in this case the BS efficiency is correspondingly reduced to 1.27, the BS/FS ratio reaches a value as high as ~12. The 2D scattering patterns are further calculated at the marked point *K* and displayed in Fig. 6d. It is directly confirmed that the FS is largely suppressed, and most of the scattered energy is radiated into the backward hemisphere with an angular beamwidth of ~92° and ~140° for the *p*-polarized and *s*-polarized components, respectively.

## Discussion

We have confirmed the possibility of using core-shell nanostructures, consisting of a metal core coated with a layer of dielectric shell having a refractive index of *n* = 2.0, to produce the ZBS or the NZFS. In most of the previous studies related to the suppression of the BS, the first Kerker condition is satisfied within the dipole approximation by overlapping the electric and magnetic dipolar modes of the same magnitude (*a*_*1*_ = *b*_*1*_ ≠ 0 and *a*_*l*_ = *b*_*l*_ = 0 for *l* ≥ 2), which requires high-permittivity dielectrics that are only achievable for limited semiconductor materials, such as silicon, germanium, and gallium arsenide^12^,^26^,^27^,^28^,^29^,^30^,^31^,^32^,^33^,^36^,^37^. Although the interplay between multipolar modes has already been proposed to suppress the BS, their excitations require specific dipole sources^24^,^33^,^34^,^35^ or the magnitudes of the specific higher order magnetic and electric modes (e.g. quadrupoles or hexapoles) are required to be equal^39^. Quite differently, in this work the ZBS is achieved under the dipole-quadrupole expansion approximation (*a*_*1*_, *a*_*2*_, *b*_*1*_, *b*_*2*_ ≠ 0 and *a*_*l*_ = *b*_*l*_ = 0 for *l* ≥ 3), where the dipolar and quadrupolar Mie coefficients, both magnetic and electric, are only required to satisfy the general condition of 3(*a*_1_ − *b*_1_) = 5(*a*_2_ − *b*_2_), i.e., they do not need to obey the constraint, such as *a*_*1*_ = *b*_*1*_ and *a*_*2*_ = *b*_*2*_ that is corresponding to the first Kerker condition^11^, 3*a*_*1*_ = 5*a*_*2*_ and 3*b*_*1*_ = 5*b*_*2*_ or *a*_*1*_ ≠ *b*_*1*_ and *a*_*2*_ = *b*_*2*_ that was proposed in^39^. Due to the involvement of the contributions from the quadrupoles, there is also no particular requirement of high-permittivity dielectrics. Well-developed wet chemical methods allow to routinely coat the silver core with a layer of low-refractive-index (*n* = 2.0) materials such as zinc sulphide or zinc oxide^43^,^44^. Based on this proposed mechanism, the ZBS with a broadband nature can be achieved and its central wavelength can be tuned from the visible to the near infrared regimes by optimizing the core-shell structural parameters. In particular, core-shell nanoparticles with identical dielectric shells but metal cores with various sizes are found to be able to effectively suppress the BS at the same wavelength, revealing a large tolerance to fabrication errors induced by the experimentally inevitable size distributions in the metal cores^45^. In addition, a broadband NZFS with a BS/FS ratio higher than 5 can also be achieved in the proposed metallo-dielectric core-shell nanostructures, when the real and imaginary parts of the Mie term 3(*a*_1_ + *b*_1_) + 5(*a*_2_ + *b*_2_) simultaneously reach minima. The above mentioned features make the proposed core-shell nanoparticles an attractive candidate for applications in nanoantennas, biosensors, and photovoltaic devices.

## Methods

The plane wave scattering by a spherical concentric core-shell nanoparticle is solved analytically using full-wave Mie theory^1^. As usually done, the incident plane wave and the scattered fields are decomposed into electric and magnetic Mie coefficients *a*_*l*_ and *b*_*l*_ (*l* is the angular moment) in a spherical coordinate system centered with the core-shell nanoparticle. By applying proper electromagnetic boundary conditions on each interface, Mie coefficients *a*_*l*_ and *b*_*l*_ can be calculated analytically. For the single-layered core-shell nanoparticles investigated in this paper, the octupolar (*a*_*3*_ and *b*_*3*_) and higher-order (*a*_*l*_ and *b*_*l*_, *l* ≥ 4) scattering are negligible in our interesting wavelength range. The BS and FS efficiencies are then computed using Eqs (4) and (5), respectively. The angular distribution of the far-field normalized scattered intensity on the two scattering planes of the azimuthal angles *φ* = 0° and 90° are calculated according to Eqs (6) and (7), respectively. In the calculations, the refractive index of the dielectric shell is assumed to be a non-dispersive value of *n* = 2.0.

## Additional Information

**How to cite this article**: Li, Y. *et al.* Broadband zero-backward and near-zero-forward scattering by metallo-dielectric core-shell nanoparticles. *Sci. Rep.* **5**, 12491; doi: 10.1038/srep12491 (2015).
